# Mental health and associated stress factors in accompanied and unaccompanied refugee minors resettled in Germany: a cross-sectional study

**DOI:** 10.1186/s13034-019-0268-1

**Published:** 2019-01-30

**Authors:** Lauritz Rudolf Floribert Müller, Karl Phillipp Büter, Rita Rosner, Johanna Unterhitzenberger

**Affiliations:** Catholic University of Eichstätt-Ingolstadt, Ostenstraße 25, 85072 Eichstätt, Germany

**Keywords:** Unaccompanied refugee minors, Asylum-seeking children and adolescents, Mental health, PTSD, Post-migration factors, Traumatic experiences, Children, European migrant crisis

## Abstract

**Background:**

Studies throughout Europe have shown that asylum-seeking children and adolescents (ASC) are at risk of developing mental disorders. The most common mental-health problems in ASC include posttraumatic stress symptoms (PTSS), internalizing symptoms such as depression and anxiety, and externalizing behaviour. Being an unaccompanied refugee minor (URM) was found to be highly predictive for higher levels of psychological distress within ASC. Nevertheless, and even though Germany is Europe’s biggest host country for ASC, studies that reliably examine the mental health of both URM and accompanied refugee minors (ARM) in Germany with psychometrically tested measures are still lacking.

**Methods:**

A cross-sectional survey in 19 facilities for minor refugees in Bavaria, Germany, screening for PTSS, depression, anxiety, externalizing behaviour, and post-migration factors was conducted. Participants were 98 ASC (URM, *n* = 68; ARM, *n* = 30) primarily from Afghanistan, Syria, and Eritrea. In 35.7% of interviews, interpreters were involved.

**Results:**

Both URM and ARM reported high levels of psychological distress and large numbers of potentially traumatic events, with 64.7% of URM and 36.7% of ARM scoring above the clinical cut-off for PTSS, 42.6% of URM and 30% of ARM for depression, and 38.2% of URM and 23.3% of ARM for anxiety. The total number of traumatic experiences was found to be the most robust predictor for PTSS, depression, and anxiety. Lower levels of individual resources, lower levels of social support in the host country, and poorer German language proficiency were associated with higher levels of psychological distress within both groups. URM reported significantly more traumatic events than ARM.

**Conclusions:**

ASC in Germany are severely distressed and burdened by the experiences of various types of potentially traumatic events. The levels of distress found in the current study correspond with rates that have been reported in previous studies with ASC throughout Europe. Limitations of the present study include the convenience sample and the cross-sectional nature of findings.

## Background

In consequence of ongoing international armed conflicts, the number of refugees, internally displaced persons, and asylum-seekers worldwide is at an all-time high: In 2017, there were more than 65 million forcibly displaced people worldwide. Of those, 22.5 million were refugees with over half of them being children and adolescent refugees under the age of 18 [1]. In the course of the so-called 2015–2017 European migrant crisis, Germany has received asylum applications from approximately 1.4 million people, resulting in Germany being Europe’s biggest host country for asylum-seekers. Almost 500,000 of them were asylum-seeking children and adolescents (ASC) under the age of 18 years [2–4]. In Germany, ASC are granted special care by the Child and Youth Welfare System (CYWS) depending on whether they enter Germany accompanied or unaccompanied. Unaccompanied refugee minors (URM)—defined as any asylum-seeking minor entering the country without the company of a person with the right of custody or guardian–normally receive specialised assistance measures in the form of accommodation in small full-care units, support by an appointed legal guardian and caregivers etc. [5]. However, these measures are restricted solely to URM and accompanied refugee minors (ARM) are not embedded in the CYWS.

There is a growing body of research suggesting that ASC show elevated rates of psychological distress [6, 7] and are at high risk for the development of serious mental disorders [8]. Posttraumatic stress symptoms (PTSS), depression, anxiety, and externalizing behaviour have been found to be the major mental health problems in this group [9–11]. A substantial number of ASC travel or seek refuge without their parents or other legal guardians. These URM have often experienced the loss of family and loved ones [12] and therefore lack the support of a family. This might negatively affect their ability to cope with stressful life events and daily stressors [13]. Accordingly, within the group of ASC, URM show the highest rates of mental health problems, exceeding the rates not only of native adolescents throughout Europe [6, 11, 14] but also of ARM [6, 7, 11, 14–16]. In several studies performed throughout Europe comparing URM and ARM, URM showed higher levels of PTSS [7, 15], depression [7, 14], and anxiety [7, 16] and reported significantly more traumatic life events [6, 15]. These findings were consistent across different types of data such as screening instruments [6], expert assessments [17], and referral records [15] and remained stable even after controlling for confounding variables such as age [6]. Furthermore, the longitudinal course of psychopathology within 1–2 years seems to be of stable nature with respect to URM [18–21].

The evidence summarized above raises the question which factors account for the increased psychopathology in ASC, and particularly in URM. From the ecological perspective suggested by Miller and Rasco [22] there are several sources of psychological distress within refugee communities. Along with results from further research particularly on ASC [23, 24], they can be roughly divided into (1) migration-related violence and trauma, (2) post-migration factors, i.e. adaptational demands regarding acculturation issues and loss, and (3) other factors that are not directly related to the experience of displacement (e.g., developmental challenges, pre-migration trauma not related to displacement).

**Fig. 1:**
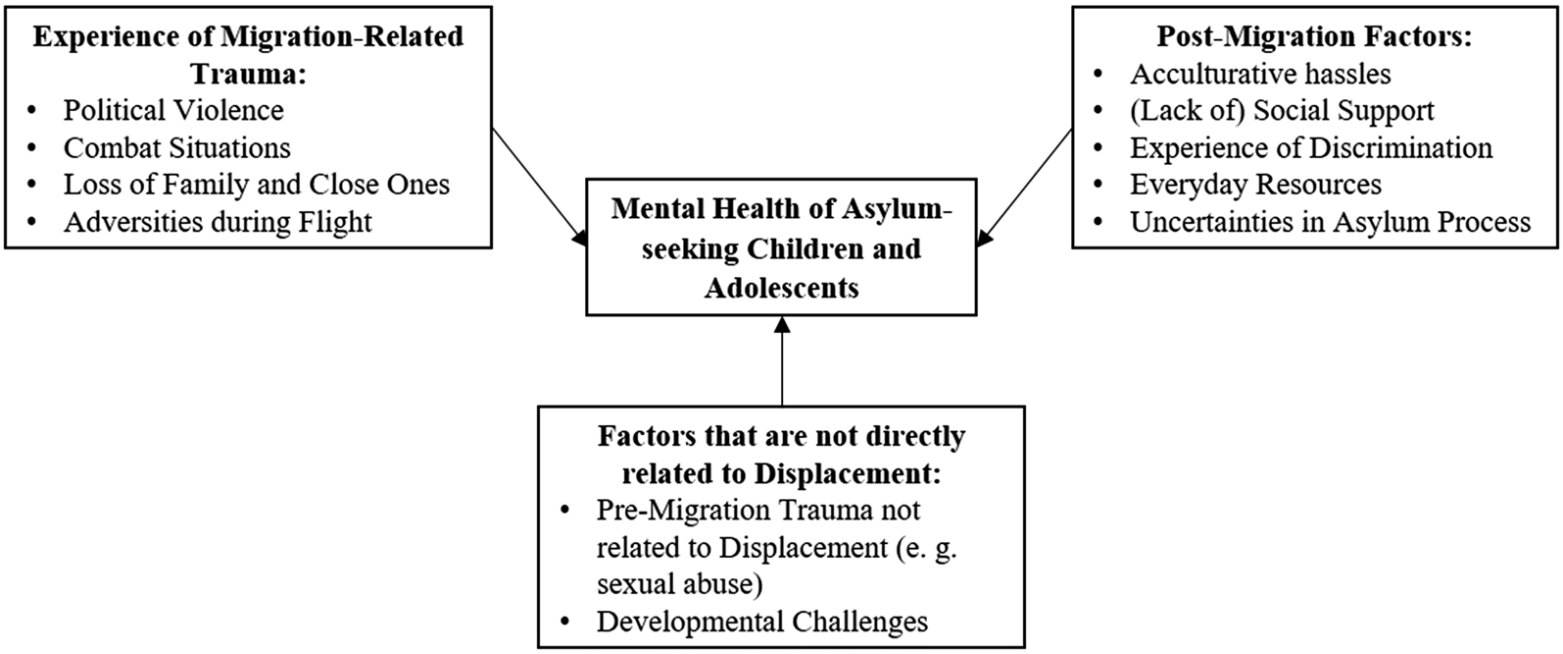
Classification of factors associated with the mental health outcome of asylum-seeking children and adolescents (ASC). The figure illustrates a dose–effect-like relationship between a multitude of potential stressors that might affect ASC’s mental health. These include experience of trauma that is related to pre- or actual migration (e.g. political violence, adversities during flight), post-migration factors that ASC are exposed to after resettlement (e.g. acculturative hassles), and factors that are not directly related to displacement (e.g. developmental challenges)

So far, research focused primarily on ASC’s migration-related trauma and PTSS [25, 26] as ASC experience a multitude of traumatic events, e.g. experience of violence, loss of family or friends, and war and combat situations [6, 12]. In fact, the total number of traumatic experiences has repetitively been found to be the most robust predictor of a poorer mental health status, exacerbating not only the levels of PTSS but also of depression and anxiety [6, 24, 27, 28]. However, in addition to traumatic events, as stated above, other individual, family, and community post-migration factors affect the severity of psychopathology in ASC [22, 24]. As yet, studies investigating the contribution of these factors have yielded mixed results: some studies showed that post-migration factors such as financial difficulties [29] and social support [30] were associated with depression only. However, there is a growing body of evidence suggesting that post-migration factors might also exacerbate levels of PTSS: Associations have been found between levels of PTSS and low-support living arrangements [12, 20, 31], refusal or insecurity of asylum [20, 29], perceived discrimination [27], and daily and acculturative stressors [21, 27], indicating the wide array of potential post-migration stressors that might affect ASC’s mental health, both in terms of depression and PTSS. Some authors argue that the cumulative effect of the above-mentioned factors (migration-related traumata and post-migration factors) in conjunction with common developmental challenges that individuals are confronted with during adolescence could contribute to the poorer overall mental health status of ASC compared to native peers [9]. Figure 1 illustrates the above-mentioned sources of psychological distress that were assembled to a classification of factors associated with the mental health outcome of ASC.

Despite the fact that high levels of psychological distress among ASC, and particularly among URM, have been consistently found in international studies, robust evidence from Germany and especially after the so-called 2015–2017 European migrant crisis investigating the mental health of URM is still scarce. To the authors’ knowledge, no standardized examination of the mental health of URM that resettled in Europe in the wake of the European migrant crisis has been conducted so far. Even latterly published reports (e.g. [20]) draw from samples that had resettled years before the recent large migration flows when some present areas of conflict had not yet emerged. Another study in a German initial reception centre examined Syrian ARM that had fled the Syrian Civil War and found Posttraumatic Stress Disorder rates of 33% in 8- to 14-years-olds [32] but did not assess URM nor post-migration factors. Experts’ reports on the management of the crisis show that the emerging demands in the areas of administration, supply, and accommodation were straining even in countries with less influx than Germany which is why the particular needs of these populations could not always be met [33]. Hence, it is key to inquire into the experience of psychological distress and post-migration factors of ASC that have resettled within the last years since current living conditions might differ from those before the crisis.

Therefore, the present study’s aims are (1) to systematically investigate for the first time the experience of trauma and levels of psychological distress among a non-utilisation sample of both URM and ARM that have arrived in Germany in the wake of the so-called 2015–2017 European migrant crisis, (2) to examine whether URM, in comparison with ARM, had experienced more traumatic events and whether they showed higher levels of psychological distress, and (3) to identify factors that might be associated with higher levels of psychopathology.

## Methods

### Procedure

**Fig. 2:**
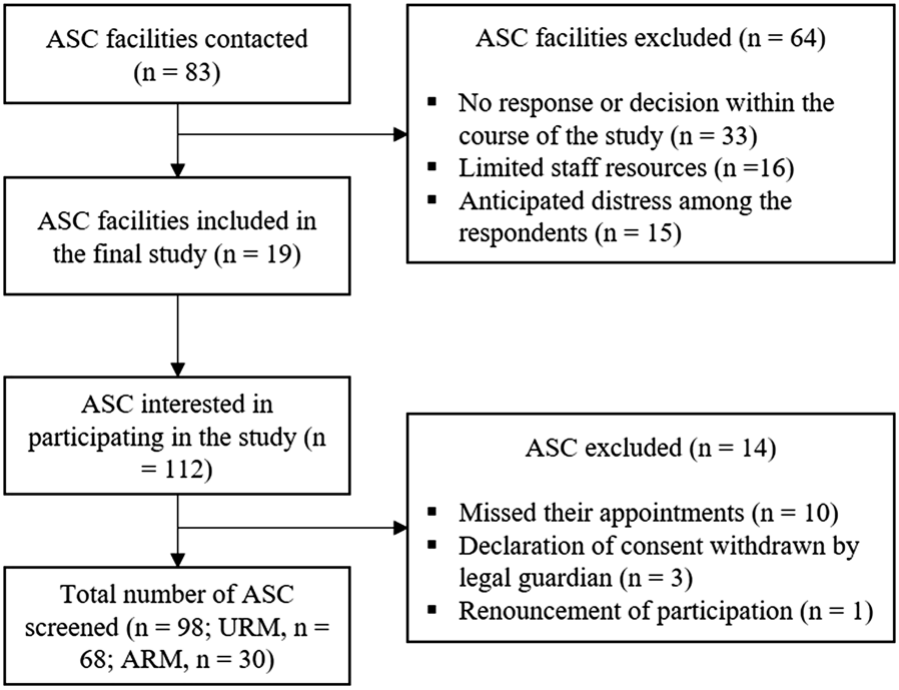
Flow of participating facilities and ASC

Participating ASC were recruited between April 2017 and September 2017. A total number of 83 ASC facilities and refugee reception centres throughout Bavaria, Germany, were contacted. In addition, the authors informed another 126 volunteers, circles of supporters, and NGOs that had expertise in the field but were no direct caregivers of ASC. These contacts were supposed to function as potential intermediaries to get in touch with the facilities where the participating ASC were living. Overall, 19 facilities agreed to support the research efforts. Most facilities that could not be obtained for participation in the study did not respond or failed to come to a decision within the course of the study (*n* = 33), others stated that their staff resources were limited and none could be spared for the survey (*n* = 16), or declined because of the anticipated distress the survey might cause among the respondents (*n* = 15). Figure 2 displays participant flow.

All facilities agreeing to participate received detailed information about the study and were asked to promote the survey among the ASC living in the respective facility. All youth wishing to participate as well as their legal guardians or parents were asked to give written informed consent including consent for publication ahead of study participation.

After recruitment, the first and second author set up appointments in the participants’ residencies to ensure the participants would feel comfortable with the setting of the inquiry. The measures were carried out in an interview-like face-to-face setting in a quiet room in each facility. All measures were administered in German but interpreters were available in case the participants did not have sufficient language competence to sufficiently understand the questions. Altogether, 35.7% of interviews were performed with involvement of interpreters. The interviews started with a clarification of the research aims, stressing, in particular, the obligation to secrecy of all involved professionals (especially researchers and interpreters), the voluntary nature of participation and the option to terminate the interview at any time. In case the interview would cause distress among the respondents the researcher was available for immediate psychological support and participants were provided with contact details of mental health services located in the area. No case of emergency was documented throughout the study. Participants received a 10-euro incentive after completion of the interview.

The study was approved by the university’s ethics committee in December 2016 (ethics approval number: 2016/23).

### Participants

One hundred and twelve ASC were recruited for participation in the study. Ten did not show up at the appointed time, in 3 cases the legal guardians withdrew their declaration of consent, and one prospective participant withdrew immediately after the initial oral information about the study. This resulted in a total study sample of 98 ASC (URM, *n* = 68; ARM, *n* = 30).

Table 1 presents the socio-demographic background of the participating ASC. The majority of participants were boys (*n* = 88, 89.9%), of Islamic faith (*n* = 81, 82.7%), lived in full-care units of the national CYWS (*n* = 66, 67.3%), and attended school in Germany (*n* = 68, 69.4%). Participants originated from 12 different countries, with Afghanistan (*n* = 54, 55.1%), Syria (*n* = 14, 14.3%), and Eritrea (*n* = 11, 11.2%) being the most common countries of origin. Most had received a decision on their asylum application, with accepted (*n* = 37, 37.8%), and rejected (*n* = 34, 34.7%) applications being similarly frequent. Another 23 (23.4%) applications were pending and data regarding asylum status were missing for four participants (4.1%). On average, participants were 16.28 (*SD* = 1.69) years of age, living in Germany for 21.46 (*SD* = 7.73) months, and had received 5.9 (*SD* = 2.93) years of schooling in their home country. All but three (*n* = 95, 96.9%) of the participants had arrived in Germany in the course of the so-called 2015–2017 European migrant crisis. These three participants (3.1%) had resettled in Germany before 2015.

**Table 1:** Sociodemographic characteristics of the participating URM and ARM

	URM, *n* = 68	ARM, *n* = 30	Total, *N* = 98	*t* test (*df*) Fisher’s exact *χ*^2^-statistics (*df*)
Age in years, *M* (*SD*)	16.78 (1.26)	15.13 (1.98)	16.28 (1.69)	*t(*39.69) = 4.2***
Gender, *n* (%)				Fisher’s exact = .001
Male	66 (97.1)	22 (73.3)	88 (89.8)	
Female	2 (2.9)	8 (26.7)	10 (10.2)	
Country of origin, *n* (%)				
Persian	30 (44.1)	25 (83.3)	55 (56.1)	*χ*^2^(1) = 13.0*** (vs. Non-Persian)
Afghanistan	29 (42.6)	25 (83.3)	54 (55.1)	
Iran	1 (1.5)	0	1 (1.0)	
Arabic	17 (25.0)	5 (16.7)	22 (22.4)	*χ*^2^(1) = .42 (vs. Non-Arabic)
Syria	12 (17.6)	2 (6.7)	14 (14.3)	
Iraq	5 (7.4)	3 (10.0)	8 (8.2)	
African	18 (26.5)	0	18 (18.4)	*χ*^2^(1) = 8.04** (vs. Non-African)
Eritrea	11 (16.2)	0	11 (11.2)	
Gambia	3 (4.4)	0	3 (3.1)	
Somalia	2 (2.9)	0	2 (2.0)	
Ethiopia	1 (1.5)	0	1 (1.0)	
Mali	1 (1.5)	0	1 (1.0)	
Others	3 (4.4)	0	3 (3.1)	
Albania	1 (1.5)	0	1 (1.0)	
Bangladesh	1 (1.5)	0	1 (1.0)	
Pakistan	1 (1.5)	0	1 (1.0)	
Religion, *n* (%)				Fisher’s exact = .001
Islam	51 (75.0)	30 (100.0)	81 (82.7)	
Others	17 (25.0)	0	17 (17.3)	
Length of stay in months, *M* (*SD*)	19.69 (5.62)	25.47 (10.16)	21.46 (7.73)	*t(*37.06) = -2.92**
Asylum status, *n* (%)				*χ*^2^(2) = 1.07
Accepted	26 (38.2)	11 (36.7)	37 (37.8)	
Rejected	21 (30.9)	13 (43.3)	34 (34.7)	
Pending	17 (25.0)	6 (20.0)	23 (23.4)	
Missing	4 (5.9)	0	4 (4.1)	

** *p* < .01, *** *p* < .001

Compared to ARM, URM were older, *t*(39.69) = 4.2, *p* < .001, had lived a shorter period of time in Germany, *t*(37.06) = − 2.92, *p* < .001, and were more likely to be male, Fisher’s exact = .001, to originate from African countries, *χ*^2^(1, *N* = 98) = 8.04, *p* < .01, and to live in residential units of the CYWS, *χ*^2^(1, *N* = 98) = 76.42, *p* < .001. ARM were more likely to be of Islamic faith, Fisher’s exact = .001, and to originate from Persian countries, *χ*^2^(1, *N* = 98) = 13.00, *p* < .001, than URM. URM and ARM did not differ with respect to further socio-demographic characteristics.

### Measures

#### Child and Adolescent Trauma Screen

Traumatic experiences and current PTSS were measured with the Child and Adolescent Trauma Screen (CATS, [34]). Firstly, participants were shown a list of 15 potentially traumatic events (CATS trauma list) and were asked to indicate whether they had ever experienced the respective traumatic event. Another four items were added to the trauma list since the original list did not contain migration-related events that ASC are likely to experience [12]. These include food deprivation, experience of dangerous journey or transport (e.g. traveling on a small crowded boat), experience of abduction, imprisonment or deportation, and committing acts of violence (voluntarily or involuntarily). Afterwards, participants rated the frequency of PTSS within the previous 2 weeks (CATS symptom scale), using 20 items on a four-point Likert scale, ranging from (0) “never” to (3) “almost always”. Finally, participants were asked to indicate if the current PTSS have impaired their everyday life within different domains by means of five dichotomous items. The PTSS score of the CATS ranges from 0 to 60 with a cut-off for clinically significant distress at 21. All PTSS according to DSM-5 are covered. The international validation of the CATS has shown good psychometric properties [34]. In the current study, the inter-item reliability of the CATS symptom scale was good (20 items; Cronbach’s α = .83).

#### Hopkins Symptom Checklist-37 for Adolescents

Symptoms of depression and anxiety, as well as externalizing behaviour, were assessed with the Hopkins Symptom Checklist-37 for Adolescents (HSCL-37A, [35]). The HSCL-37A is a prolonged version of the original HSCL-25 [36]. Participants rate the frequency of 37 symptoms within the last 4 weeks by means of a four-point Likert scale, ranging from (1) “not/never” to (4) “always”. All 37 items sum up to a total score, ranging from 37 to 148 points, indicating global psychological distress. Subscales for depression (15 items), anxiety (10 items), internalizing symptoms (the sum of the “depression” and the “anxiety” scale, 25 items), and externalizing behaviour (12 items) can be calculated. The HSCL-37A has no set clinical cut-off levels but some authors have suggested using percentile scores derived from research with URM in Belgium as indicators for the need for psychosocial intervention [28, 35]. These criteria were used in the present study and are referred to as clinical cut-off values. They are as follows: Total score, 69 points; internalizing symptoms, 54 points; depression, 33 points; anxiety, 20 points (all 60th percentile); externalizing behaviour, 19 points (90th percentile). The HSCL 37-A is a commonly used measure to screen for internalizing symptoms and externalizing behaviour and is widely used among ASC populations (e.g., [18, 19]). It has been interculturally validated and shows good psychometric properties [35]. In the current study, the inter-item reliability of the total score (α = .88), the depression (α = .83), anxiety (α = .83), and the internalizing subscales (α = .89) was good. Inter-item reliability of the externalizing subscale was not satisfactory (α = .53).

#### Everyday Resources and Stressors Scale

Levels of resources and stressors in participants’ everyday lives were examined using the Everyday Resources and Stressors Scale (ERSS, Büter and Müller, unpublished scale). The ERSS is a 20-items self-report questionnaire developed to screen for the following post-migration factors: (a) experience of discrimination; (b) social support within the family, (c) social support in the host country, (d) language proficiency, and (e) everyday resources. Respondents are asked to rate their experience of each item using four-point Likert scales [1–4]. The questionnaire was composed by means of construction and aggregation of items to screen for relevant post-migration factors identified through literature recommendations [24, 37]. One subscale was derived from the Everyday Discrimination Scale [38]. In the current study, the inter-item reliability of the subscales was as follows: discrimination (4 items; α = .77), social support in the host country (3 items; α = .71), the social support within the family (5 items; α = .75), language proficiency (3 items; α = .73), everyday resources (5 items; α = .71).

### Statistical analyses

Data were analysed using IBM SPSS statistics, version 25. To test for differences between groups with respect to categorical data, *χ*^2^-statistics were used, using Fisher’s exact tests for expected cell sizes below five in two by two contingency tables. To test for mean differences between groups with respect to continuous data, *t*-tests were used for equal groups and Welch’s *t*-tests for unequal groups, with a set level of significance of .05; in all cases using the Holm-Bonferroni method to control for multiple comparisons. Group differences were examined using ANCOVAs, with socio-demographic data as independent variables and mental health outcome measures as dependent variables (CATS trauma list, CATS symptom scale, and HSCL-37A). In order to avoid small sample sizes, countries of origin were merged into four categories (Persian, Arabic, African, and other countries). “Other countries of origin” was excluded from analysis due to the small cell size. Effects of gender and accommodation type could not be tested due to insufficient subsample sizes and insufficient within-group (URM vs. ARM) variance, respectively. In the case of the CATS trauma list, group (URM or ARM) and country of origin were entered as fixed factors, with age as covariate. In the case of the CATS symptom scale and the HSCL-37A measures, fixed factors were group (URM or ARM), country of origin, and asylum status, with total number of traumatic events, age, and length of stay as covariates. Bivariate Pearson correlations were used to examine associations between continuous socio-demographic data, ERSS scores, and mental health measures. Finally, multiple stepwise hierarchical regression analyses were carried out to identify significant predictors of CATS trauma list, CATS symptom scale, and HSCL-37A scores. In case of CATS symptom scale and HSCL-37A scores, predictor variables included in the analyses were CATS trauma list, socio-demographic data, and ERSS scores. In case of CATS trauma list, predictor variables included in the analysis were socio-demographic data and ERSS scores.

## Results

### Experience of trauma and levels of psychological distress

#### Trauma

An overview of the experience of specific traumatic events is given in Table 2.

**Table 2:** Experience of trauma amongst URM and ARM, derived from the CATS trauma list

Item (did you ever experience …)	URM, *N* = 68	ARM, *N* = 30	Total, *N* = 98	χ^2^-statistics (*df*) Fisher’s exact
	*n* (%)	*n* (%)	*n* (%)	
Dangerous journey or transport	66 (97.1)	29 (96.7)	95 (96.6)	Fisher’s exact = 1
Seeing someone in the community get slapped, punched or beat up	57 (83.8)	20 (66.7)	77 (78.6)	Fisher’s exact = .06
Lack of food or water for several days	57 (83.8)	18 (60)	75 (76.5)	Fisher’s exact = .02*
Seeing someone attacked, stabbed, shot at, hurt badly or killed	55 (80.9)	20 (66.7)	75 (76.5)	Fisher’s exact = .2
Being around war	49 (72.1)	14 (46.7)	63 (64.3)	5.85 (1)*
Someone close to you dying suddenly or violently	51 (75)	10 (33.3)	61 (62.2)	15.38 (1)***
Slapped, punched or beat up by someone not in the family	43 (63.2)	15 (50)	58 (59.2)	1.73 (1)
Serious accident or injury	46 (67.6)	8 (26.7)	54 (55.1)	14.13 (1)***
Seeing someone in the family get slapped, punched or beat up	30 (44.1)	16 (53.3)	46 (46.9)	.61 (1)
Attacked, stabbed, shot at or hurt badly	35 (51.5)	9 (30)	44 (44.9)	3.88 (1)*
Being robbed by threat, force or weapon	30 (44.1)	13 (43.3)	43 (43.9)	.005 (1)
Slapped, punched or beat up in the family	28 (41.2)	11 (36.7)	39 (39.8)	.23 (1)
Imprisonment or abduction	28 (41.2)	7 (23.3)	35 (35.7)	3.06 (1)
Serious natural disaster	18 (26.5)	10 (33.3)	28 (28.6)	Fisher’s exact = .63
Scary medical procedure	18 (26.5)	6 (20)	24 (24.5)	Fisher’s exact = .61
Committing acts of violence (voluntarily or involuntarily)	9 (13.2)	4 (13.3)	13 (13.4)	Fisher’s exact = 1
Someone older touching your private parts when they shouldn’t	9 (13.2)	2 (6.7)	11 (11.2)	Fisher’s exact = .50
Someone forcing or pressuring sex	3 (4.4)	2 (6.7)	5 (5.1)	Fisher’s exact = .64

* *p* < .05, *** *p* < .001

In total, numbers of traumatic events were high: All participants had experienced at least one traumatic event and on average, participants reported 8.82 different traumatic experiences (*SD* = 2.99, range 1–15). The most frequently reported traumatic experience was the migration-related event “dangerous journey or transport (e.g. traveling on a small crowded boat or in the trunk of a car)” (96.6%). In addition, some 75% of participants had witnessed someone in the community get slapped, punched or beat up (78.6%), attacked, stabbed, shot at, hurt badly or killed (76.5%), and had experienced the lack of food or water for several days (76.5%). The least frequently reported traumatic experience was “someone forcing or pressuring sex” (5.1%). What is more, experience of interpersonal violence (either within the family or elsewhere, 85.7%), war (64.3%), and loss (62.2%) were also highly prevalent.

#### Mental health outcomes

Table 3 provides an overview of mental health outcomes for both URM and ARM.

**Table 3:** Means, standard deviations, cut-offs, and mean comparisons of groups for the CATS and HSCL-37A measures

	URM, *N* = 68		ARM, *N* = 30		Total, *N* = 98		ANCOVA main effect group *F (df)*^a^
	*M* (*SD*)	Subjects above cut-off *n* (%)	*M* (*SD*)	Subjects above cut-off *n* (%)	*M* (*SD*)	Subjects above cut-off *n* (%)	
CATS-TL	9.49 (2.7)		7.3 (3.12)		8.82 (2.99)		10.15 (1, 89)***
CATS-SS	23.69 (8.75)	44 (64.7)	19.47 (10.14)	11 (36.7)	22.4 (9.35)	55 (56.1)	.04 (1, 75)
HSCL-Tot	66.66 (12.86)	26 (38.2)	59.83 (11.89)	7 (23.3)	64.57 (12.9)	33 (33.7)	.99 (1, 75)
HSCL-Dep	31.5 (7.67)	29 (42.6)	28.4 (7.15)	9 (30)	30.55 (7.61)	33 (33.7)	1.05 (1, 75)
HSCL-Anx	19.21 (5.4)	26 (38.2)	17.3 (5.34)	7 (23.3)	18.62 (5.43)	38 (38.8)	.08 (1, 75)
HSCL-Int	50.71 (12.27)	23 (33.8)	45.7 (10.62)	7 (23.3)	49.17 (11.96)	30 (30.6)	.26 (1, 75)
HSCL-Ext	15.96 (2.78)	7 (10.3)	14.13 (1.99)	1 (3.3)	15.4 (2.69)	8 (8.2)	4.75 (1, 75)*

*CATS-TL* CATS trauma list, *CATS-SS* CATS symptom scale, *HSCL-Tot* HSCL-37A total score, *HSCL-Dep* HSCL-37A depression scale, *HSCL-Anx* HSCL-37A anxiety scale, *HSCL-Int* HSCL-37A internalizing cluster, *HSCL-Ext* HSCL-37A externalizing cluster

^a^ Covariates (CATS-TL): age; covariates (CATS-SS and HSCL-37A measures): age, total number of traumatic events, length of stay

* *p* < .05, *** *p* < .001

In all, 55 participants (56.1%) scored above the clinical cut-off on the CATS symptom scale, indicating need for psychosocial intervention for PTSS. When evaluated according to DSM-5 criteria, 29.6% of participants fulfilled diagnostic criteria for PTSD.

With respect to the HSCL-37A measures, some 30 participants scored above the clinical cut-off values on the respective scales: 33 participants (33.7%) on the total scale, 30 participants (30.6%) on the internalizing scale, 38 participants (38.8%) on the anxiety scale, 33 participants (33.7%) on the depression scale, and 8 participants (8.2%) on the externalizing scale.

### Group differences

#### Experience of trauma

URM reported significantly more traumatic experiences than ARM, even when controlling for age, *F* (1, 89) = 10.15, *p* = .001. *χ*^2^-statistics comparing groups yielded differences in the experience of specific traumatic events: Thus, URM were more likely to have experienced “someone close to you dying suddenly or violently”, *χ*^2^(1, *N* = 98) = 15.38, *p* < .001, a “serious accident or injury”, *χ*^2^(1, *N* = 98) = 14.13, *p* < .001, “being around war”, *χ*^2^(1, *N* = 98) = 5.85, *p* < .05, the “lack of food or water for several days”, Fisher’s exact = .02, and getting “attacked, stabbed, shot at or hurt badly”, *χ*^2^(1, *N* = 98) = 3.88, *p* < .05.

#### Mental health outcomes

URM (64%) were more likely to score above the cut-off value for PTSS than ARM (36%), *χ*^2^(1, *N* = 98) = 6.65, *p* < .05, but no significant effect was found when controlling for total number of traumatic events, age, and length of stay in the subsequent three-way analysis of covariance with CATS symptom scale as dependent variable, *F* (1, 75) = .04, *p* = n. s.

With regard to the HSCL-37A scores, after controlling for total number of traumatic events, age, and length of stay, being unaccompanied was found to be related to the externalizing subscale, *F* (1, 75) = 4.75, *p* = .032, such that URM showed significantly higher scores than ARM, *t*(75.93) = 3.67, *p* < .001. Being unaccompanied was not found to be related to any other of the HSCL-37A measures.

### Predictors of experience of trauma and mental health outcomes

The results of the bivariate Pearson correlations are given in Table 4, the results of the multiple hierarchical regressions analysis are given in Table 5.

**Table 4:** Correlations between experience of traumatic events, demographic data, post-migration factors, and mental health outcomes for 98 ASC

Variable	CATS-TL	CATS-SS	HSCL-Tot	HSCL-Dep	HSCL-Anx	HSCL-Int	HSCL-Ext
CATS-TL	1	.50***	.47***	.46***	.35***	.45***	.28**
Age	.37***	.16	.13	.13	.10	.13	.04
Length of stay	− .13	− .24*	− .12	− .11	− .06	− .10	− .14
Number of residents in facility	− .19	− .14	− .20*	− .25*	− .08	− .20	− .10
Years of schooling	− .07	− .22*	− .14	− .12	− .13	− .13	− .05
ERSS discrimination	.11	.19	.19	.20*	.12	.18	.10
ERSS social support within family	− .20*	− .21	− .17	− .18	− .16	− .18	− .01
ERSS social support in host country	− .04	− .19	− .22*	− .16	− .28**	− .23*	− .03
ERSS language proficiency	.03	− .29**	− .22*	− .23*	− .11	− .19	− .18
ERSS resources	− .16	− .39***	− .39***	− .33**	− .28**	− .33**	− .39***

*CATS-TL* CATS trauma list, *CATS-SS* CATS symptom scale, *HSCL-Tot* HSCL-37A total score, *HSCL-Dep* HSCL-37A depression scale, *HSCL-Anx* HSCL-37A anxiety scale, *HSCL-Int* HSCL-37A internalizing cluster, *HSCL-Ext* HSCL-37A externalizing cluster

* *p* < .05, ** *p* < .01, *** *p* < .001

**Table 5:** Hierarchical regression analysis for variables predicting CATS and HSCL-37A measures

Step	Included Variable	Stand. β^a^	*F* change	Overall *F*	*df*	Adj. *R*^2^ change	Total adj. *R*^2^
CATS-TL							
Step 1	Age	.248	12.401***	12.401***	1, 95	.115	.115
Step 2	Group (URM or ARM)	.21	3.944*	8.365***	2, 94	.036	.151
CATS-SS							
Step 1	Number of traumatic events	.457	30.56***	30.56***	1, 95	.235	.235
Step 2	ERSS resources	− .254	14.01***	24.38***	2, 94	.092	.327
Step 3	ERSS language proficiency	− .231	7.59**	19.92***	3, 93	.045	.372
HSCL-Tot							
Step 1	Number of traumatic events	.405	25.08***	25.08***	1, 95	.201	.201
Step 2	ERSS resources	− .33	14.62***	21.645***	2, 94	.10	.301
HSCL-Dep							
Step 1	Number of traumatic events	.429	25.288***	25.288***	1, 95	.182	.20
Step 2	ERSS resources	− .21	8.666**	17.987***	2, 94	.059	.259
Step 3	ERSS language proficiency	− .185	4.28*	13.832***	3, 93	.025	.284
HSCL-Anx							
Step 1	Number of traumatic events	.323	11.955***	11.955***	1, 95	.102	.102
Step 2	ERSS social support in host country	− .263	7.953**	10.391***	2, 94	.062	.164
HSCL-Int							
Step 1	Number of traumatic events	.386	21.507***	21.507***	1, 95	.176	.176
Step 2	ERSS resources	− .275	9.382**	16.393***	2, 94	.067	.243
HSCL-Ext							
Step 1	ERSS resources	− .361	17.28***	17.28***	1, 95	.143	.144
Step 2	Group (URM or ARM)	.274	9.121**	13.931***	2, 94	.067	.211

*CATS-TL* CATS trauma list, *CATS-SS* CATS symptom scale, *HSCL-Tot* HSCL-37A total score, *HSCL-Dep* HSCL-37A depression scale, *HSCL-Anx* HSCL-37A anxiety scale, *HSCL-Int* HSCL-37A internalizing cluster, *HSCL-Ext* HSCL-37A externalizing cluster

^a^ For the final model/step

* *p* < .05, ** *p* < .01, *** *p* < .001

#### Trauma

Two-way analysis of covariance revealed a significant main effect for region of origin, *F* (1, 87) = 3.71, *p* = .015, but post hoc testing yielded no significant differences between different countries of origin in experience of trauma. Moreover, the total number of traumatic experiences was significantly correlated with age, *r*(96) = .45, *p* < .001, and social support within the family, *r*(96) = − .20, *p* < .05.

The subsequent hierarchical regression analysis demonstrated that age, *β* = .25, *t*(94) = 2.35, *p* < .05, and being unaccompanied, *β* = .21, *t*(94) = 1.99, *p* < .05, significantly predicted the total number of traumatic experiences, accounting for a significant proportion of variance in the CATS trauma list, *R*^*2*^_*adj*_ = .15, *F*(2, 94) = 8.37, *p* < .001.

#### Mental health outcomes

Three-way analyses of covariance with age, length of stay, and number of traumatic experiences as covariates and CATS symptom scale and HSCL-37A scores as dependent variables did not yield any significant effects regarding country of origin and asylum status.

CATS symptom scale was significantly correlated with total number of traumatic experiences, *r*(96) = .50, *p* < .001, everyday resources, *r*(96) = − .39, *p* < .001, language proficiency, *r*(96) = − .29, *p* < .01, and social support within the family, *r*(96) = − .21, *p* < .05. All but social support within the family also significantly predicted PTSS scores in a subsequent regression analysis, accounting altogether for 37% of variance in the CATS symptom scale, *R*^*2*^_*adj*_ = .37, *F*(3, 93) = 19.92, *p* < .001.

As can be seen in Table 4, total number of traumatic experiences and everyday resources were significantly correlated with all HSCL-37A measures. In addition to that, all ERSS measures–except social support within the family–as well as further socio-demographic variables were significantly correlated with at least one HSCL-37A measure.

Finally, regression analysis demonstrated that the included predictor variables accounted for significant proportions of variance in all HSCL-37A measures. Total number of traumatic experiences was the strongest predictor for all HSCL-37A measures, except for the externalizing scale. Total number of traumatic experiences and everyday resources significantly predicted the total score, *R*^*2*^_*adj*_ = .30, *F*(2, 94) = 21.65, *p* < .001, and the internalizing scale, *R*^*2*^_*adj*_ = .24, *F*(2, 94) = 16.93, *p* < .001; total number of traumatic experiences, everyday resources, and language proficiency significantly predicted the depression scale, *R*^*2*^_*adj*_ = .28, *F*(3, 93) = 13.83, *p* < .001; and total number of traumatic experiences and social support within the host country significantly predicted the anxiety scale, *R*^*2*^_*adj*_ = .16, *F*(2, 94) = 10.39, *p* < .001. The externalizing scale was significantly predicted by everyday resources and being unaccompanied, *R*^*2*^_*adj*_ = .21, *F*(2, 94) = 13.93, *p* < .001.

## Discussion

The present study examined the rates of traumatic experiences and levels of psychological distress, including PTSS, depression, anxiety, and externalizing behaviour, in a sample of 98 ASC resettled in Germany between 2015–2017. To the authors’ knowledge, it is the first study in Germany investigating the mental health of both URM and ARM in a standardized manner and the first at all after the so-called 2015–2017 European migrant crisis.

As expected, the results indicate the high prevalence of traumatic experiences and severity of psychological distress among ASC. Migration-related traumatic events were found within the most frequently reported traumatic experiences, but also traumatic events not necessarily related to migration (e.g. serious accident or injury) were highly prevalent. The reported experience of loss (62.2%) was comparable with other studies that examined both URM and ARM (45.7% to 69.55% [6, 12]); but experience of war trauma (64.3%) in this sample was higher when compared to these studies (34.51% to 41.9%). Conceivably, these differences might be due to the different measures applied and the sample composition. Samples of young refugees are heterogeneous with differing distributions of countries depending on current areas of conflict and developments worldwide. The levels of PTSS (56.1%) and depression (33.7%) found in this sample were in the upper range of most studies that examined both URM and ARM and reported their findings in terms of percentages [12, 27, 29]. In these studies, levels of PTSS above the clinical cut-off ranged from 19 to 54% and levels of depression ranged from 3 to 30%. Yet, none of these studies assessed levels of anxiety and externalizing behaviour. When compared descriptively to the sample described by Bean and colleagues [6], the means of anxiety in the present sample were similar in URM and slightly higher in ARM. The latter could be due to the fact that the ARM sample in their study also included immigrant adolescents without a history of flight. Arguably, these youths might show lower levels of distress than ARM and thus, the overall ‘accompanied’ subsample in this study might be somewhat skewed. Except for the externalizing scale, the same applies for the further HSCL-37A measures. In accordance with previous studies using the HSCL-37A, externalizing problems were not found to be major problems that ASC reportedly struggle with [6, 28]. It could be that ASC tend to respond to severe adversities in a rather internalizing manner. However, it seems also plausible that ASC underreport externalizing behaviour because they might worry about the possibly negative consequences on their asylum process. Furthermore, the inter-item reliability of the HSCL-37A externalizing scale was not satisfactory, so results regarding externalizing behaviour should be interpreted cautiously. It is noteworthy that this scale has previously been found to show the lowest inter-item reliability of all HSCL-37A scales, ranging at the edge of a satisfactory *α*-value [6, 19].

The comparison between the URM and ARM groups revealed mixed results: In accordance with previous studies, URM reported significantly more traumatic experiences than ARM [6, 12], even after taking age into account. Moreover, URM were more likely to experience a number of specific traumatic events, stressing once again the increased vulnerability of URM towards experience of trauma, both related (e.g. “being around war”) and not directly related to migration (e.g. “serious accident or injury”). In terms of psychopathology, however, the present study yielded results that are contrary to previous studies [6, 12]. In absolute terms, URM showed higher means in all measures of psychopathology, but this difference was found to be significant only with respect to externalizing behaviour. Arguably, this could be due to the small sample size, resulting in a slightly insufficient test power when set for a moderate effect size of Cohen’s *d* of .5. Apart from that, it is also possible that URM benefit from the high professional support they receive within the CYWS, resulting in comparable levels of psychological distress though having experienced more traumatic events than ARM. As opposed to ARM, only URM in Germany are granted special support actions by the CYWS (e.g. full-care housing) that aim at meeting their particular needs [5, 39]. ARM, on the other hand, typically live with their parents or other guardians, who may be struggling with mental health issues and post-migration stressors themselves. Indeed, some studies have demonstrated the negative effects of parental psychiatric problems on ASC’s mental health [40, 41].

With regard to different factors possibly associated with the mental health of ASC described in Fig. 1, traumatic experiences, socio-demographic data, and post-migration factors were analysed as predictors for the outcome measures. Consistent with other studies on the impact of trauma on ASC’s mental health (for an overview, see [24]), the total number of traumatic experiences was found to be the most robust predictor for a poorer mental health status. Total number of traumatic experiences was predictive for all symptom scales assessed with exception of externalizing symptoms. The proportions of variance accounted for by the total number of traumatic experiences ranged from 10.2% (anxiety) to 23.5% (PTSS). Thus, targeting the experience of trauma in psychotherapy might arguably also mitigate symptoms of depression and anxiety and thus ameliorate ASC’s overall mental health status [42]. After taking trauma exposure into account, a number of post-migration factors also contributed to the levels of psychological distress in ASC. Most importantly, everyday resources were predictive for all symptom scales except anxiety. With regard to externalizing behaviour, having more everyday resources were even found to be the major predictor of lower levels of symptoms. This is in line with results suggesting that active coping strategies are associated with reduced risk for externalizing and internalizing problems [43]. These activities (like practicing sports, meeting friends) might function as positive coping strategies and could contribute to reducing levels of symptoms. What is more, language proficiency was found to account for significant proportions of variance in PTSS and depression scores and social support in the host country for significant proportions of variance in anxiety scores. It is noteworthy that these are domains that are directly linked to ASC’s integration into the host country. Unexpectedly, ASC did not differ in symptom severity depending on their asylum status. Again, this is most probably due to insufficient test power because differences fell just short of statistical significance and the constituent subgroups were relatively small.

These findings are in accordance with various results from other research on ASC that demonstrated the predictive quality of post-migration factors in addition to trauma exposure [12, 27]. In this way, they support the above-mentioned classification of factors associated with the mental health outcome in ASC (see Fig. 1). They also underline the importance of an increased sensitivity of professionals in contact with ASC not only for possible preceding trauma experiences but also for post-migration factors that might affect ASC’s mental health.

### Strengths and limitations

Strengths: First, to the authors’ knowledge, this is the first study in Germany examining and comparing the experience of trauma, psychological distress, and post-migration factors in both URM and ARM and the first at all after the so-called 2015–2017 European migrant crisis. As both URM and ARM were included, direct comparisons between these two groups could be carried out. Second, standardized measures were employed that are widely used among minor refugee populations, making it possible to compare the results with those reported in previous studies. Third, the authors did not focus on mental health outcomes alone but assessed post-migration factors, as well. Lastly, ASC were assessed in an interview-like setting with attendance of interpreters which resulted in a close-to-zero extent of missing data because difficulties in understanding could be solved.

As well as strengths, this study has several limitations. Most importantly, it is necessary to acknowledge that the composition of the sample might result in a number of distortions: First, the sample is not representative and bias may have occurred on the level of both institutions and individuals. The vast majority of institutions did not respond to the recruitment efforts, which makes it possible that the ASC included in the study lived in environments with especially good resources. One can also assume that ASC with the highest levels of distress did not participate as they or their caregivers did not want to risk increasing symptoms by answering questions on trauma and PTSS. Attrition also occurred on the basis of retracted decision processes, including the personnel not wanting to take responsibility for decision-making or their insecurities about whether they were legally allowed to carry out the survey in their premises. Therefore, further research on representative samples (as in [6]) should be carried out, preferably fostered by the state to dispel any of the above-mentioned insecurities. Second, the study sample did not include a sufficient number of girls to further analyse differences based on gender. The same is evident for effects of accommodation type on ASC’s mental health as almost all URM were living in full-care units whereas almost all ARM were living in settings without further care. On the other hand, the present sample reflects the composition of ASC in Germany, where most refugees are young males. Moreover, screening measures were used to determine levels of only the most common mental health problems among ASC. Thus, findings are reported in terms of cut-off values which are merely an estimate of psychopathology. Diagnostic interviews should be used in further research with ASC to determine, on the one hand, more reliably the prevalence of certain diagnoses and to cover, on the other hand, the wide range of possible mental health problems that ASC struggle with.

Moreover, the assessment of psychopathology and demographics relied solely on ASC’s self-report. Further sources of information, especially with regard to demographic data such as the asylum status, might have resulted in even more reliable data.

Finally, the data are of cross-sectional nature which precludes causal assertions. More longitudinal research should be added to the handful of studies that examined the course of symptoms among ASC.

## Conclusions

The findings of the current study indicate that psychopathology among ASC in Germany is severe and seems to substantially exceed those of native youths [44, 45]. Even though the overall living conditions of this population—because of the overburdening of the authorities during the 2015–2017 European migrant crisis—might arguably be inferior to those of ASC that had resettled earlier, the levels of psychological distress were similar to those found in previous studies throughout other European countries. Since this study is the first to report on both URM and ARM that resettled in the course of the European migrant crisis it brings forward first insights into the mental health status and associated stress factors among this vulnerable population that might function as a connecting point for further research on representative samples and treatment approaches. As such, it adds to the aspiration of depicting the as-is state in a hard-to-reach population, as well as deriving appropriate actions for improvement of symptoms. Even though numbers of asylum applications in Europe are declining [46], researchers, clinicians, and policy-makers must not make the mistake of suspending dedication towards this topic as, up to now, no European country has accomplished to provide a satisfactory care system for ASC. Hitherto, only a small percentage of ASC gain access to mental health care [47] and, despite the decline in Europe, numbers of refugees worldwide are most likely to further increase on account of various reasons such as climate change [48].

Taken together, this study has several clinical implications. The results reemphasise the need for mental health services in general and trauma-focused treatment in particular, as traumatic experiences seem to affect a whole spectrum of mental health problems among ASC. Given the fact that only a small percentage of ASC with clinically relevant symptoms receive treatment, the results evoke the urgent need for authorities to take actions to provide appropriate approaches for meeting the psychological needs of ASC. Besides guaranteeing that mental health interventions be financed, further training of social workers and professional caregivers is crucial. With training in trauma-informed care and psychological screenings, we can achieve an enhanced sensitivity for ASC’s mental health problems. As ASC seem to internalize their problems and may show low levels of functional impairment, their mental health problems might remain undiscovered by caregivers [28]. Apart from their capacity as contact persons to initiate appropriate treatment social workers can, as a next step, be trained to conduct preventive support groups for ASC with sub-clinical symptoms [49]. As a last step, trauma-focused treatments are necessary to address those with clinical PTSS. As trauma-focused cognitive behavioural therapy (TF-CBT [50]) has been shown to significantly reduce PTSS and symptoms of depression and anxiety [42], it could improve ASC’s overall mental health status. A case series examining TF-CBT for URM in Germany [51] has shown promising results for the feasibility of this evidence-based treatment for PTSD in refugee youths. Nevertheless, research regarding the effectiveness of psychosocial interventions for ASC is still scarce [52].

At the moment, ARM in Germany and most other European countries have even fewer possibilities to gain access to a stepped-care approach like the one described above (screening, prevention, intervention) than URM. Mostly living with their families, they do not routinely come into contact with social workers or other professionals who might detect mental health problems and make a referral. Therefore, it is paramount that ARM be integrated into the CYWS or a comparable support system since ARM, too, show high levels of psychological distress.

Besides their role of guidance into appropriate treatment, the CYWS should engineer a structure that empowers ASC to develop positive coping strategies. More resources like playing sports and meeting friends were associated with lower levels of most mental health outcomes. In light of this fact, it seems crucial to evaluate and strengthen ASC’s individual sources of possible resources as these could function as positive coping strategies and thus mitigate their symptoms.

## Abbreviations

ARM: accompanied refugee minors
ASC: asylum-seeking children and adolescents
CATS: Child and Adolescent Trauma Screen
CYWS: Child and Youth Welfare System
ERSS: Everyday Resources and Stressors Scale
HSCL-37A: Hopkins Symptom Checklist-37 for Adolescents
PTSD: posttraumatic stress disorder
PTSS: posttraumatic stress symptoms
TF-CBT: trauma-focused cognitive behavioural therapy
URM: unaccompanied refugee minors
